# A Study on the Diagnostic and Prognostic Value of Extrachromosomal Circular DNA in Breast Cancer

**DOI:** 10.3390/genes16070802

**Published:** 2025-07-06

**Authors:** Fuyu Li, Wenxiang Lu, Lingsong Yao, Yunfei Bai

**Affiliations:** State Key Laboratory of Digital Medical Engineering, School of Biological Science and Medical Engineering, Southeast University, Nanjing 210096, China

**Keywords:** extrachromosomal circular DNA, eccDNA, breast cancer, diagnostic model, prognostic evaluation

## Abstract

Objectives: To investigate the clinical diagnostic and prognostic value of extrachromosomal circular DNA (eccDNA) in breast cancer, eccDNA profiles were constructed for 81 breast cancer tumor tissues and 33 adjacent non-tumor tissues. Methods: The distribution characteristics of eccDNA across functional genomic elements and repetitive sequences were systematically analyzed. Furthermore, a diagnostic model for differentiating malignant and normal breast tissues, as well as a prognostic prediction model, was developed using a random forest algorithm. Results: EccDNA in breast cancer tissues harbor a higher proportion of functional elements and repetitive sequences, with their annotated genes significantly enriched in tumor- and immune-related pathways. However, no significant differences in eccDNA features were observed across breast cancer subtypes or pathological stages. In the validation cohort, the eccDNA-based diagnostic model achieved an AUC of 0.83, with repetitive elements and enhancer-associated features contributing the most to diagnostic performance. The prognostic model achieved an AUC of 0.78, with repetitive element annotations also showing strong prognostic relevance. Conclusions: These findings highlight the promising potential of eccDNA in the development of precision diagnostics and prognostic systems for breast cancer.

## 1. Introduction

Breast cancer is one of the most common malignant tumors affecting women, posing a serious threat to women’s health and placing a substantial burden on society and national healthcare systems. Early screening, prevention, and treatment are critical for optimizing the clinical management of the disease [1]. Therefore, identifying biomarkers with higher early-warning value, greater accuracy, and broader applicability has long been a major focus in breast cancer research.

Extrachromosomal circular DNA (eccDNA) refers to circular DNA molecules that exist independently of chromosomes [2,3]. Studies have shown that eccDNA can promote tumorigenesis and cancer progression through various mechanisms, including oncogene amplification, enhanced transcriptional activity, increased tumor heterogeneity, and the facilitation of drug resistance [4,5,6,7,8,9,10,11]. Due to their structural stability and widespread distribution, eccDNA demonstrate strong potential for use in tissue and liquid biopsies and are increasingly recognized as a promising class of novel molecular biomarkers with significant clinical potential [12,13].

In breast cancer, eccDNA may influence tumor development by encoding functional genes. Human epidermal growth factor receptor 2 (*ERBB2*/HER2) is a critical indicator for molecular subtyping and a key therapeutic target in breast cancer [14,15]. It has been reported that up to 30% of amplified *ERBB2* genes were encoded in eccDNA, and these eccDNA often co-amplify enhancer elements to accelerate disease progression [9,16]. Yang et al. identified eccDNA carrying amplified *TRPS1*, whose overexpression promotes malignant transformation and drives genomic rearrangements and therapy resistance in breast cancer [17]. EccDNA encoding *MIR6748* can upregulate miR-6748, thereby suppressing the tumor suppressor gene *TUSC5* and promoting breast cancer invasion and progression [18,19]. Our previous work also revealed that eccDNA harboring gene fragments such as *FAT2* and *CTNNB1* serve as potential prognostic biomarkers for predicting outcomes and overall survival in patients with invasive breast cancer [20]. These findings collectively provide a theoretical basis for the application of eccDNA encoding functional genomic elements in precision diagnosis, prognostic evaluation, and targeted therapy.

In addition to functional genomic elements, eccDNA are also closely associated with genomic repetitive sequences. On the one hand, the formation of eccDNA often relies on homologous recombination or microhomology-mediated repair mechanisms involving repetitive sequences [21,22]. On the other hand, eccDNA frequently carry repetitive elements, especially in tumor cells, including Alu families, long interspersed nuclear elements (LINEs), and telomeric sequences [23,24]. Mammalian-wide interspersed repeats (MIRs) represent one of the oldest transposable element families in the human genome. Enhancer elements derived from MIRs are characterized by highly open chromatin structures and abundant transcription factor binding sites, enabling them to regulate gene expression through transcription factor recruitment [25].

To date, multiple studies have developed tumor diagnostic and prognostic models based on eccDNA-encoded genes, confirming the diagnostic and prognostic significance of eccDNA genomic features [26,27,28]. Our previous research has also demonstrated that the distribution patterns of genomic repetitive elements within eccDNA possess strong diagnostic potential for breast cancer [29], although their prognostic value still needs to be fully elucidated. Building upon this foundation, this present study further explores the features of eccDNA-encoded functional elements, such as genes and enhancers, and integrates them with repetitive sequence element profiles to systematically characterize eccDNA landscapes in tumor tissues and matched adjacent normal tissues from clinical breast cancer samples. By incorporating transcriptomic and immunohistochemical phenotype data, we further investigated the eccDNA characteristics across different subtypes of breast cancer. Ultimately, machine learning algorithms were employed to construct both diagnostic models for distinguishing benign and malignant breast tissues and prognostic models for disease-free survival (DFS). This work identifies eccDNA features with significant diagnostic and prognostic value, and the systematic quantification of eccDNA features can contribute to the development of personalized medicine for breast cancer.

## 2. Materials and Methods

### 2.1. Research Samples

This retrospective study was conducted using clinical samples obtained from Jiangsu Provincial People’s Hospital, with approval from the hospital’s ethics committee (approval ID: 2019SR512). All the patients provided informed consent before enrollment. All samples were collected between 2016 and 2018 from female patients with histologically confirmed invasive ductal carcinoma, comprising a total of 81 breast cancer tissue samples and 33 matched adjacent normal tissue samples. Immunohistochemical (IHC) analysis was performed on the tumor samples to evaluate the expression of the estrogen receptor (ER), progesterone receptor (PR), and HER2, as well as the Ki-67 proliferation index. The IHC subtypes were defined according to the following criteria: samples that were ER-positive, HER2-negative, and had high PR expression (>20%) and a low Ki-67 index (<20%) were classified as Luminal-A breast cancer; samples that were ER-positive, HER2-negative, and had either low PR expression (<20%) or a high Ki-67 index (>20%) were classified as Luminal-B; samples that were ER- and HER2-positive were also classified as Luminal-B; samples that were negative for ER and PR but positive for HER2 were classified as HER2-enriched; and samples that were negative for ER, PR, and HER2 were classified as triple-negative breast cancer (TNBC). The clinical characteristics of the 81 cases are summarized in Table 1.

### 2.2. EccDNA Enrichment and Sequencing

Total DNA was extracted using the QIAamp DNA Mini Kit (Qiagen, Hilden, Germany). Following extraction, linear DNA was digested using the restriction enzyme PacI (New England Biolabs, Ipswich, MA, USA) in combination with Plasmid-Safe ATP-dependent DNase (Epicentre, Madison, WI, USA). Residual linear DNA was further removed using a circular DNA-specific purification kit. The purified eccDNA was then subjected to rolling circle amplification using Phi29 DNA polymerase (New England Biolabs, Ipswich, MA, USA) [30,31]. The amplification products were used for library construction according to the instructions of the OnePot Pro DNA Library Prep Kit (Yeasen, Shanghai, China) and were subsequently subjected to paired-end sequencing (150 bp read length) on the Illumina NovaSeq 6000 platform (Illumina, San Diego, CA, USA) (Table S1).

### 2.3. EccDNA Identification and Annotation

Sequencing data were aligned to the human reference genome GRCh38 using BWA-MEM (v0.7.17-r1188) with default parameters [32]. Identification of eccDNA was performed using the Circle-Map tool with the Realign module [33], and candidate eccDNA were filtered based on the following criteria: (1) at least 2 supporting split reads; (2) a total of at least 3 supporting split reads or discordant read pairs; (3) a Circle-Map score ≥ 50; (4) the proportion of internal sequencing depth at breakpoints > 0.33; and (5) the proportion of uncovered bases being within the eccDNA region < 0.1.

Genomic coordinates of identified eccDNA were intersected with various genomic elements using Bedtools intersect (v2.29.2) to annotate functional and repetitive elements [34,35]. Gene, exon, intron, and untranslated region (UTR) annotations were obtained from the GENCODE database (v27, Ensembl 90) [36]. Intergenic regions were calculated based on gene annotations and chromosome lengths, while gene upstream and downstream regions were defined as 2000 base pairs upstream from gene start sites and 2000 base pairs downstream from gene end sites, respectively. Breast cancer enhancer annotations were derived from EnhancerDB, specifically from the MCF7 breast cancer cell line dataset [37]. CpG island and DNase I hypersensitive site (DHS) data were downloaded from the UCSC Genome Browser, with upstream and downstream regions defined similarly as 2000 bp flanking the CpG island boundaries. Repetitive sequence information was also obtained from the UCSC Genome Browser via the RepeatMasker annotation file, including short interspersed nuclear elements (SINEs), LINEs, long terminal repeat elements (LTRs), DNA repeat elements, simple repeats, low-complexity repeats, and satellite repeats [38,39]. In addition, annotation statistics were separately calculated for Alu and MIR families, as well as for transposons.

The results of eccDNA identification and annotation on breast cancer tissue and noncancerous adjacent tissue are summarized in Table S1.

### 2.4. Genomic Features of eccDNA

Based on the distribution patterns of eccDNA across functional genomic elements and repetitive sequences, a set of quantitative features was designed in this study. These features include the total number of eccDNA per million mapped sequencing reads; the number and proportion of eccDNA annotated to genes, enhancers, and DHS regions; the proportions of eccDNA mapped to exons, introns, UTRs, upstream and downstream gene regions, CpG islands and their flanking regions; the number and proportion of eccDNA annotated to all repetitive elements; as well as the proportions of eccDNA associated with six major classes of genomic repetitive elements and specific categories such as Alu, MIRs, and transposons. In total, 58 quantitative features were extracted for further analysis.

### 2.5. Transcriptome Analysis

Gene expression quantification was performed using HTSeq (v0.13.5) to generate a gene-level count matrix for each sample [40,41]. Breast cancer molecular subtypes were determined using the R package genefu (v 2.22.1) based on the PAM50 classifier, categorizing samples into Luminal-A, Luminal-B, HER2-enriched, Basal-like, and Normal-like subtypes [42]. Differentially expressed genes (DEGs) among subtypes were identified using analysis of variance (ANOVA), with significance thresholds defined as a standard deviation (SD) > 2 and a false discovery rate (FDR) < 0.05 [43]. Pathway enrichment analysis of DEGs was conducted using clusterProfiler (v4.6.2), focusing on Kyoto encyclopedia of genes and genomes (KEGG) enrichment and gene ontology (GO) enrichment in the biological process category, with adjusted *p*-values < 0.05 considered statistically significant [44].

### 2.6. Prognostic Analysis

Based on the genomic characteristics of eccDNA, the optimal cutoff method was used to determine high and low feature values, thereby enabling the binary labeling of each feature. The Kaplan–Meier method was employed to evaluate survival differences associated with each binarized eccDNA feature, with DFS as the clinical endpoint [45]. Statistical significance was assessed using the log-rank test, and a *p*-value < 0.05 was considered indicative of a significant difference.

### 2.7. Construction of eccDNA-Based Diagnostic and Prognostic Prediction Model

This study compared the classification performance of five different algorithms: random forest, Logistic Regression, Naïve Bayes, a support vector machine (SVM), and K-nearest neighbors (KNNs). The dataset was split into training and testing sets at a 2:1 ratio, and model training was performed using ten-fold cross-validation. Hyperparameter optimization was conducted via grid search to maximize model performance. Evaluation metrics included sensitivity, specificity, precision, F1 score, accuracy, and the area under the receiver operating characteristic curve (AUC).

## 3. Results

### 3.1. EccDNA Landscape in Breast Tumor and Adjacent Non-Tumor Tissues

**Figure 1:**
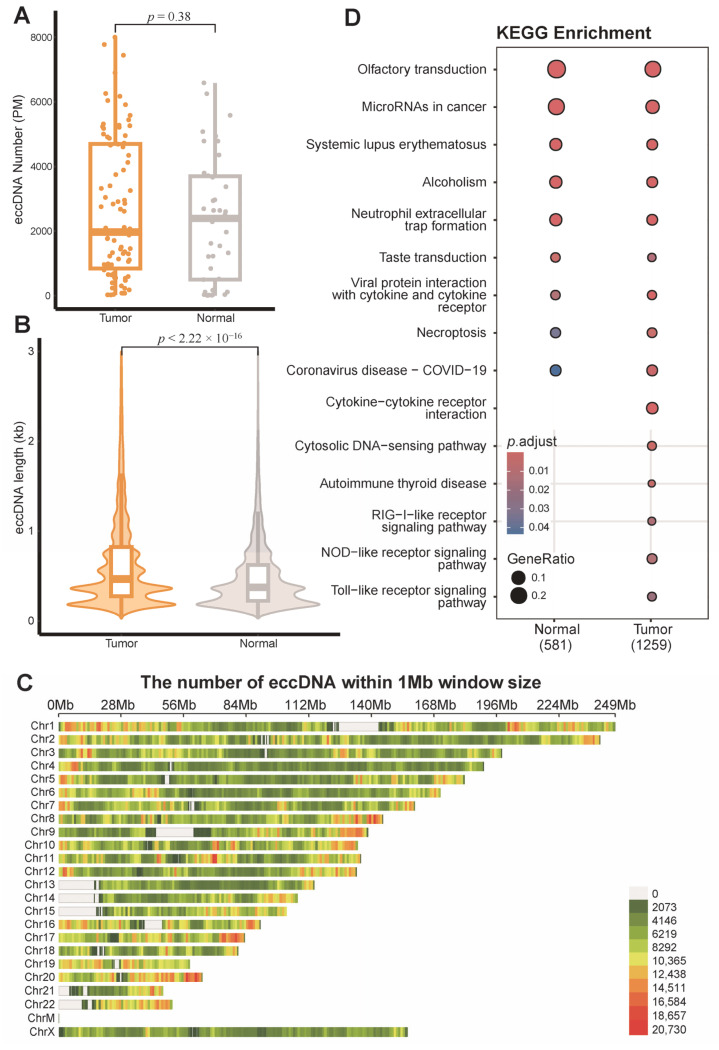
Extrachromosomal circular DNA (eccDNA) profiles of breast cancer tissues and matched adjacent normal tissues. (**A**) Box plot of eccDNA numbers per million mapped reads (*Wilcoxon* test, *p* = 0.38). (**B**) The size distribution of eccDNA (*Wilcoxon* test, *p* < 2.22 × 10^−16^). (**C**) Genome-wide distribution of eccDNA numbers in 1 Mb genomic windows across breast cancer tissues. (**D**) The Kyoto encyclopedia of genes and genomes (KEGG) pathway enriched by eccDNA-annotated genes.

A total of 19,727,489 eccDNA molecules were identified across 81 breast tumor tissue samples, and 8,333,324 eccDNA were identified in 33 matched adjacent non-tumor tissue samples. The number of eccDNA varied considerably among individual samples; the eccDNA burden ranged from 2.77 to 8000.25 per million mapped reads. The overall eccDNA abundance showed no significant difference between tumor and adjacent non-tumor tissues (Figure 1A). However, eccDNA in tumor tissues exhibited a significantly longer average length (835.135 bp, *p* < 2.2 × 10^−16^), with a distinct bimodal length distribution (peaks at approximately 180 bp and 360 bp; Figure 1B), compared to adjacent tissues. This shift toward longer eccDNA in tumors may be associated with microhomology-mediated circularization favored by genomic instability, suggesting that selective pressures within the tumor microenvironment may promote the retention of functionally relevant, larger eccDNA.

The results revealed a pronounced chromosomal preference in the distribution of eccDNA in breast cancer (Figure S1). Specifically, the proportion of eccDNA in tumor tissues was significantly higher than that in adjacent normal tissues on chromosomes 1, 8, 17, 19, and 20, while significantly lower levels were observed on chromosomes 3, 4, 5, 6, 12, 13, 18, and the X chromosome (*p* < 0.05). In tumor samples, eccDNA hotspot regions were found to overlap with known breast cancer risk gene clusters [46]. For instance, the eccDNA density in the chr17q12-q21 region reached as high as 14,511 per megabase (Mb), where key oncogenes such as *ERBB2*/HER2 (Chr17:39688087–39746718), *GRB7* (Chr17:39750021–39775543), and *TOP2A* (Chr17:35241156–35281915) are located. The eccDNA burden in this region was significantly higher than in non-hotspot regions (Figure 1C and Figure S2). Moreover, eccDNA were markedly enriched at chromosomal termini, suggesting that these regions, due to their inherent genomic instability, enrichment of repetitive sequences, and replication stress, may serve as hotspots for eccDNA generation.

Gene annotation analysis showed that 75.18% of eccDNA originated from genic regions, and the genes carried by these eccDNA were enriched in multiple breast cancer–associated pathways, including olfactory transduction, microRNAs in cancer, and systemic lupus erythematosus, as identified by KEGG pathway analysis. These findings suggest that eccDNA may contribute to tumor progression through the epigenetic regulation and dysregulation of immune responses (Figure 1D). Furthermore, GO enrichment analysis indicated that eccDNA-encoded genes may promote breast cancer metastasis via the regulation of angiogenesis (Figure S3).

**Figure 2:**
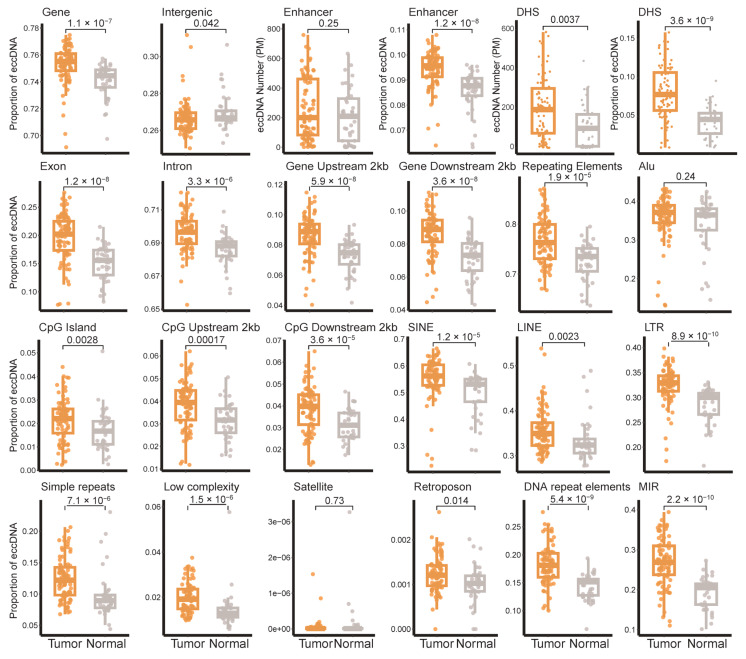
Comparative distribution of extrachromosomal circular DNA (eccDNA) across genomic elements in breast tumor versus adjacent normal tissues. *p*-values are determined using the Wilcoxon test. Orange point, tumor tissue sample; gray point, adjacent normal tissue sample. PM, per million mapped reads; DHS, DNase I hypersensitive site; SINE, short interspersed nuclear elements; LINE, long interspersed nuclear elements; LTR, long terminal repeat elements; MIR, Mammalian-wide interspersed repeat.

We further compared the distribution characteristics of eccDNA across genomic elements between breast tumor tissues and adjacent non-tumor tissues (Figure 2). The proportion of eccDNA annotated to functional genomic elements was significantly higher in tumor samples, particularly in enhancers, exons, upstream gene regions, CpG islands, and their flanking regions (*p* < 0.01). Notably, eccDNA annotated to DHSs were also significantly enriched in tumor tissues (*p* < 0.001). Given that DHSs represent open chromatin regions and transcription factor binding hotspots, the strong association between eccDNA and DHSs suggests that tumor-derived eccDNA are more likely to carry active regulatory elements, potentially conferring increased transcriptional activity.

Beyond functional elements, we also assessed the annotation of eccDNA with respect to repetitive genomic sequences. Except for satellite repeats, eccDNA derived from SINEs, LINEs, LTRs, DNA repeat elements, simple repeats, and low-complexity repeats were all significantly more abundant in tumor tissues compared to adjacent non-tumor tissues (Figure 2). Additionally, we specifically analyzed eccDNA originating from Alu and MIR families as well as transposons. While the Alu-related eccDNA distribution did not show significant differences between tissue types, despite Alu elements being recognized markers for cancer progression and prognosis, the abundance of MIR-derived eccDNA was markedly elevated in tumor tissues (*p* < 0.001).

### 3.2. Distribution of eccDNA Across Breast Cancer Subtypes

Breast cancer is a highly heterogeneous disease, and the PAM50 molecular classification is considered the gold standard for its complex subtype stratification, providing critical guidance for clinical treatment decisions. Based on gene expression profiling, the 81 breast cancer tissue samples were classified into 20 Luminal-A, 19 Luminal-B, 14 HER2-enriched, 22 Basal-like, and 6 Normal-like cases (Figure S4). Integrating differential gene expression analysis with eccDNA gene annotation results, a total of 390 eccDNA-associated genes were found to be significantly differentially expressed among subtypes (Figure S4). Of these, 198 genes encoded by subtype-specific eccDNA were identified, with the majority enriched in Luminal-A and Basal-like subtypes (Figures S5 and S6). Notably, eccDNA specific to the Luminal-A subtype were predominantly annotated to genes enriched in the estrogen signaling pathway, consistent with its clinical IHC phenotype (Figure S7). Furthermore, when comparing tumor and adjacent normal tissues, no statistically significant differences were observed in the genomic distribution features of eccDNA among different PAM50 molecular subtypes, clinical IHC subtypes, or pathological stages (Figures S8–S10).

### 3.3. Breast Cancer Diagnostic Model Based on eccDNA Features

**Figure 3:**
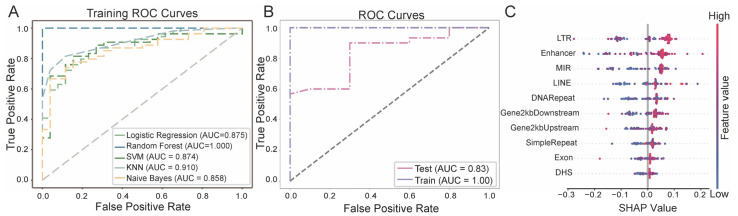
Performance of breast cancer diagnostic model based on extrachromosomal circular DNA (eccDNA) features. (**A**) Receiver operating characteristic (ROC) curves of all models on the training data. (**B**) ROC curves of the random forest model on the training and test data. (**C**) Feature importance based on Shapley Additive Explanation (SHAP) analysis of the random forest model. AUC, area under curve; SVM, support vector machine; KNN, K-nearest neighbors; DHS, DNase I hypersensitive site; LINE, long interspersed nuclear elements; LTR, long terminal repeat elements; MIR, Mammalian-wide interspersed repeat.

A total of 58 features were extracted based on the proportional distribution of eccDNA within functional genomic elements and repetitive sequences to construct a diagnostic model for breast cancer. The performance of five different predictive algorithms was compared, and the random forest model was selected as the optimal diagnostic model based on its AUC performance in the training set (Figure 3A). The random forest model achieved an AUC of 0.83 in the test set, indicating a comparable discriminatory capacity for breast cancer diagnosis based on eccDNA features (Figure 3B).

To enhance the interpretability of the model, Shapley Additive Explanation (SHAP) was employed to assess the contribution of ten key features to the prediction outcomes (Figure 3C). Among these, the proportion of eccDNA annotated to LTRs exhibited the highest importance. The proportions of eccDNA derived from other repetitive sequence elements, including MIRs, LINEs, DNA repeats, and simple repeats, were also significantly positively correlated with tumor status. Given that eccDNA originating from repetitive elements is often associated with genomic instability, these findings suggest that repeat-derived eccDNA may promote tumorigenesis by inducing genomic instability. Additionally, the enrichment of eccDNA annotated to functional regulatory elements, such as enhancers and 2 kb upstream/downstream gene regions, in tumor samples implies a potential oncogenic mechanism whereby eccDNA disrupts transcriptional regulatory networks. In contrast, eccDNA associated with exonic regions showed SHAP value distributions tightly clustered around zero, indicating minimal contribution to model predictions.

### 3.4. EccDNA Features as Indicators of DFS in Breast Cancer

**Figure 4:**
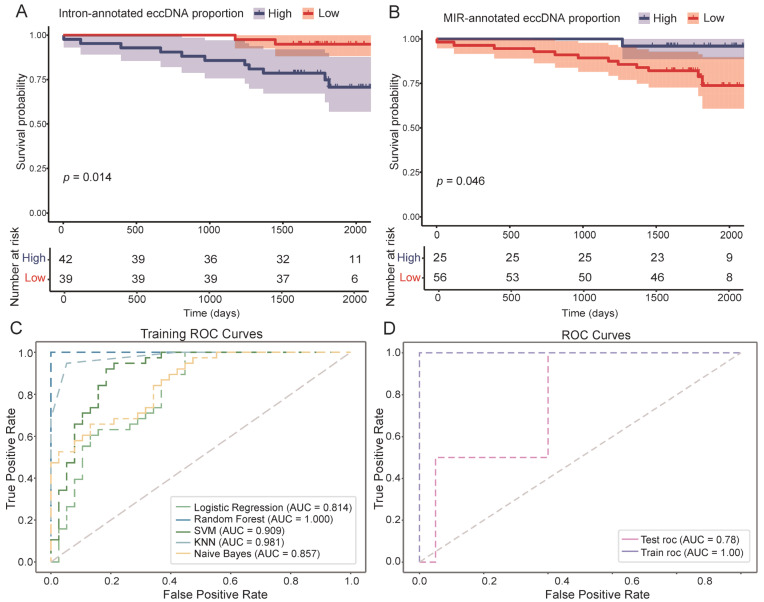
Survival analysis and prognostic prediction model for disease-free survival (DFS) based on extrachromosomal circular DNA (eccDNA) features in breast cancer. (**A**) Kaplan–Meier curves of intron-annotated eccDNA features. (**B**) Kaplan–Meier curves of Mammalian-wide interspersed repeat (MIR)-annotated eccDNA features. (**C**) Receiver operating characteristic (ROC) curves of all models on the training data. (**D**) ROC curves of random forest model on the training and test data. AUC, area under curve; SVM, support vector machine; KNN, K-nearest neighbors.

Among the 81 breast cancer patients included in this study, 13 experienced disease recurrence or death, while 10 were lost to follow-up; the remaining patients were free from recurrence or death at the end of the follow-up period. Based on survival analysis and statistical testing, nine eccDNA-related features were found to be significantly associated with breast cancer DFS (*p* < 0.05). Specifically, patients with a high proportion of eccDNA annotated to intronic regions and genomic repetitive elements tended to have poorer prognoses (Figure 4A). In contrast, higher proportions of eccDNA annotated to exons, upstream gene regions, intergenic regions, and DHSs were generally associated with more favorable outcomes (Figure S11). Notably, although a high overall proportion of eccDNA originating from repetitive genomic sequences was identified as a risk factor for breast cancer, eccDNA features derived from specific repeat elements, including LINEs, MIRs, and DNA repeats, were found to be protective factors when present at high levels (Figure 4B). Also, subtype-specific analysis revealed that prognostically relevant eccDNA features were identified in luminal-A (satellite and simple-repeat-annotated eccDNA proportion, *p* < 0.05) and luminal-B (DHS-annotated eccDNA proportion, *p* < 0.05) breast cancers (Figure S12), while no significant associations were observed in the HER2-enriched and TNBC subtypes, likely due to limited sample sizes.

Additionally, the univariate Cox proportional hazards analysis of eccDNA features revealed that a lower proportion of intron-annotated eccDNA (hazard ratio = 0.185, 95% confidence interval: 0.0410–0.838, *p* = 0.0285) and repeat-annotated eccDNA (hazard ratio = 0.268, 95% confidence interval: 0.0737–0.977, *p* = 0.0459) was significantly associated with improved clinical outcomes, suggesting their potential as protective prognostic indicators in breast cancer. In contrast, several eccDNA features demonstrated hazard ratios greater than six, indicating a possible association with poorer prognosis (Table 2). However, these associations did not reach statistical significance. Overall, these findings suggest that while certain eccDNA annotation categories may have prognostic relevance, further validation in larger, independent cohorts is required to confirm their clinical utility.

Furthermore, we constructed a prognostic prediction model for breast cancer based on eccDNA features using the random forest algorithm. The model achieved an AUC of 0.78 on the test set, demonstrating the potential of eccDNA features as prognostic biomarkers for DFS and their clinical utility in breast cancer (Figure 4C,D).

## 4. Discussion

In this work, based on 81 clinical breast cancer tissue samples and 33 matched adjacent normal tissue samples, we comprehensively investigated the differences in eccDNA characteristics between tumor and non-tumor tissues, including length distribution, the annotation across functional genomic elements, chromatin accessibility, and genomic repeat elements. The findings provide further evidence that eccDNA participates in and influences tumor development and progression through multiple mechanisms.

On the one hand, genes encoded by eccDNA in tumor tissues are significantly enriched in multiple pathways related to tumor development and immune responses, including olfactory transduction, autoimmune thyroid disease, and microRNAs in cancer. These genes are involved in various processes such as proliferation, invasion, angiogenesis, and the metastasis of breast cancer [47,48,49,50]. On the other hand, eccDNA in both tumor and adjacent normal tissues encodes a range of inflammation- and immune-related genes, including those involved in the neutrophil extracellular trap formation pathway and systemic lupus erythematosus [51,52,53]. Notably, eccDNA from tumor tissues showed specific enrichment in immune pathways such as the cytosolic DNA-sensing and Cytokine−cytokine receptor interaction pathways [54,55,56,57].

Additionally, a higher proportion of eccDNA in tumor samples were annotated to enhancer regions and open chromatin regions, highlighting their potential roles in transcriptional regulation. EccDNA derived from various genomic repeat elements, particularly MIRs and transposons, were also more abundant in tumor tissues. This suggests that eccDNA may cooperate with repeat elements to drive genomic instability and oncogenesis. For instance, the aberrant activation of transposons can induce chromosomal double-strand breaks or genomic rearrangements, promoting eccDNA formation; in turn, eccDNA with encoded transposons can reintegrate into the genome or replicate independently, further exacerbating genomic instability and epigenetic dysregulation. Collectively, the annotation results suggest that eccDNA may contribute to breast cancer progression through oncogene amplification, the modulation of immune responses, and the induction of genomic instability.

To explore the clinical value of eccDNA, we constructed diagnostic and prognostic models for breast cancer. The diagnostic model, based on the top-10 important eccDNA features, achieved comparable performance in distinguishing tumor tissue from normal tissue (AUC = 0.83 in the test set), while the prognostic model reached an AUC of 0.78 for predicting DFS events, underscoring the potential of eccDNA as a molecular biomarker for both diagnosis and prognosis. Feature importance analysis further suggested that indirect regulatory roles of eccDNA, such as mediating chromatin remodeling or carrying regulatory elements, may be more prevalent and biologically relevant than direct oncogene amplification in the context of breast cancer development.

Interestingly, we found no significant differences in eccDNA distribution patterns across breast cancer PAM50 molecular subtypes, clinical IHC subtypes, or pathological stages. This suggests that the localization characteristics of tumor-derived eccDNA may reflect general genomic instability in cancer cells, rather than subtype- or stage-specific features.

However, this study has certain limitations. On the one hand, the sample size of the study cohort is relatively small, and the diagnostic and prognostic value of eccDNA features requires further validation in larger clinical cohorts of breast cancer patients. Moreover, the performance of the diagnostic and prognostic models could be further improved in the future through more refined feature engineering. On the other hand, the functional mechanisms by which eccDNA contributes to breast cancer initiation and progression still need to be validated through biological experiments. To address these limitations, future work could focus on expanding the study cohort by incorporating multicenter clinical datasets or publicly available breast cancer cohorts to improve statistical power and generalizability. Additionally, applying advanced feature engineering techniques, such as representation learning or multimodal integration, may enhance the performance of diagnostic and prognostic models. Furthermore, functional validation through in vitro and in vivo experiments is essential to elucidate the biological roles of eccDNA in tumorigenesis and progression.

## 5. Conclusions

This study systematically constructed a comprehensive eccDNA landscape in breast cancer tumor tissues and matched adjacent normal tissues. EccDNA in breast cancer tissues were significantly enriched in transcriptionally active regions and carried a higher proportion of functional genomic elements and repetitive sequences, suggesting that they may promote tumor progression through hijacking regulatory elements and inducing genomic instability. Furthermore, diagnostic and prognostic models based on eccDNA features were developed, demonstrating comparable discrimination between malignant and non-malignant tissues as well as predictive power for DFS events. These findings provide new insights into the potential clinical utility of eccDNA in breast cancer diagnosis and prognosis.

## Figures and Tables

**Table 1:** Statistics on clinical information of 81 breast cancer tumor tissue samples.

Information		Sample Number (81 in Total)
Age		25~68 (Mean: 48.7)
ER status	Positive	56
	Negative	25
PR status	Positive	50
	Negative	31
HER2 status	Positive	14
	Negative	67
Ki-67 status	High	63
	Low	18
Immunohistochemical subtype	Luminal-A	14
	Luminal-B	44
	HER2-enriched	7
	Triple-negative	16
Pathological stage	I	26
	II	45
	III	10

ER, estrogen receptor; PR, progesterone receptor; HER2, human epidermal growth factor receptor 2.

**Table 2:** Hazard ratios (95% confidence interval) of eccDNA annotation features.

eccDNA Feature	Hazard Ratios (95% Confidence Interval)	*p*-Value
Intron-annotated eccDNA proportion	0.185 (0.0410–0.838)	0.0285
Repeat-annotated eccDNA proportion	0.268 (0.0737–0.977)	0.0459
MIR-annotated eccDNA proportion	6.17 (0.801–47.6)	0.0806
DNA repeat-annotated eccDNA proportion	6.10 (0.790–47.2)	0.0829
Exon-annotated eccDNA proportion	6.29 (0.817–48.5)	0.0775
Gene Upstream-annotated eccDNA proportion	6.20 (0.805–47.8)	0.0797
LINE-annotated eccDNA proportion	7.17 (0.930–55.4)	0.0587

eccDNA, extrachromosomal circular DNA; MIR, mammalian-wide interspersed repeat; LINE, long interspersed nuclear element.

## Data Availability

The data presented in this study are available in the National Genomics Data Center with the accession numbers HRA007650, HRA007696, and HRA007678.

## Supplementary Materials

The following supporting information can be downloaded at https://www.mdpi.com/article/10.3390/genes16070802/s1: Figure S1: Chromosomal distribution of eccDNA; Figure S2: Genome-wide distribution of eccDNA; Figure S3: The GO pathway enriched by eccDNA-annotated genes; Figure S4: Heatmap of differentially expressed genes across breast cancer PAM50 subtypes; Figure S5: Volcano plots of differentially expressed genes and subtype-specific eccDNA-annotated genes across breast cancer PAM50 subtypes; Figure S6: Heatmap of differentially expressed subtype-specific eccDNA-annotated genes across breast cancer PAM50 subtypes; Figure S7: KEGG enrichment of differentially expressed subtype-specific eccDNA-annotated genes across breast cancer PAM50 subtypes; Figure S8: Comparative distribution of eccDNA across genomic elements and repeat sequences in breast cancer subtypes; Figure S9: Comparative distribution of eccDNA across genomic elements and repeat sequences in breast cancer IHC subtypes; Figure S10: Comparative distribution of eccDNA across genomic elements and repeat sequences in breast cancer samples with different clinical stages; Figure S11: Kaplan–Meier curves of seven eccDNA features for DFS in breast cancer; Figure S12: Kaplan–Meier curves of three subtype-specific eccDNA features for DFS. Table S1: Summary of eccDNA annotation and sequencing on breast cancer tissue and noncancerous adjacent tissue.

## Abbreviations

The following abbreviations are used in this manuscript:

eccDNA	Extrachromosomal circular DNA
*ERBB2*/HER2	Human epidermal growth factor receptor 2
DFS	Disease-free survival
LINE	Long interspersed nuclear element
SINE	Short interspersed nuclear element
MIR	Mammalian-wide interspersed repeat
LTR	Long terminal repeat element
ER	Estrogen receptor
PR	Progesterone receptor
UTR	Untranslated region
DHS	DNase I hypersensitive site
ROC	Receiver operating characteristic
AUC	Area under the receiver operating characteristic curve
SVM	Support vector machine
KNN	K-nearest neighbors
DEG	Differentially expressed gene
ANOVA	Analysis of variance
SD	Standard deviation
FDR	False discovery rate
GO	Gene Ontology
KEGG	Kyoto encyclopedia of genes and genomes
