# Supplementary material for: A Study on the Diagnostic and Prognostic Value of Extrachromosomal Circular DNA in Breast Cancer
Source: Genes (Basel). 2025 Jul 6;16(7):802. doi: 10.3390/genes16070802 (PMC12295733; doi:10.3390/genes16070802)
Supplement: Supplementary file 1 [file genes-16-00802-s001.zip › genes-3729268-supplementary.pdf]

A Study on the diagnostic and prognostic value of extrachromosomal circular DNA in breast cancer

Fuyu Li, Wenxiang Lu, Lingsong Yao and Yunfei Bai*

State Key Laboratory of Digital Medical Engineering, School of Biological Science and Medical Engineering, Southeast University, Nanjing, 210096, China; 230198834@seu.edu.cn (F.L.); luwen-xiang@seu.edu.cn (W.L.); yaolsyy@foxmail.com (L.Y.)

* Correspondence: Yunfei Bai, PhD; State Key Laboratory of Digital Medical Engineering, School of Biological Science and Medical Engineering, Southeast University; Tel.: +86-25-83790500; Fax: +86-025-83362442; Email: whitecf@seu.edu.cn

Supplementary Materials

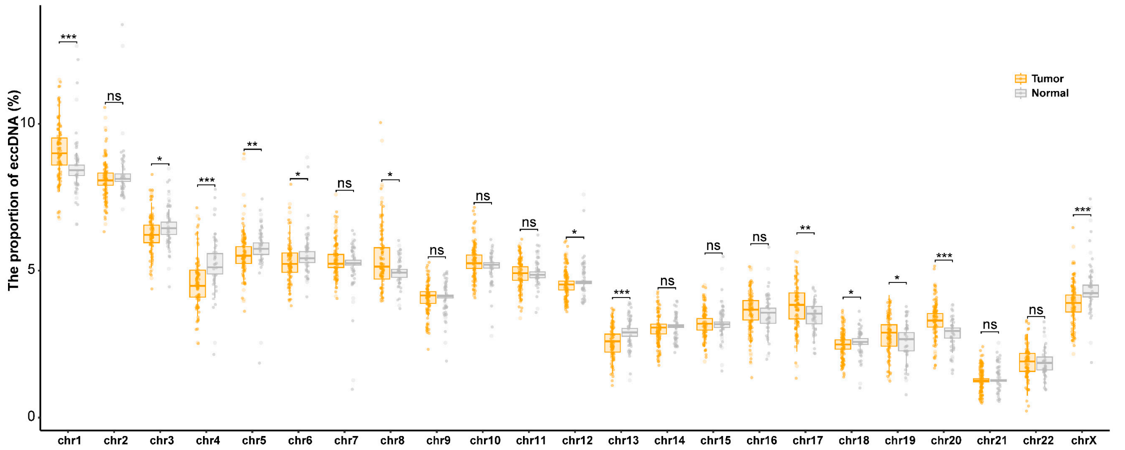

Figure S1: Chromosomal distribution of eccDNA proportions in 81 breast cancer tissues (yellow box plots) and 33 matched normal tissues (gray box plots). p-values are determined using the Wilcoxon test. Significance: (***) p-value<0.001, (**) p-value<0.01, (*) p-value<0.05, (ns) No significance.

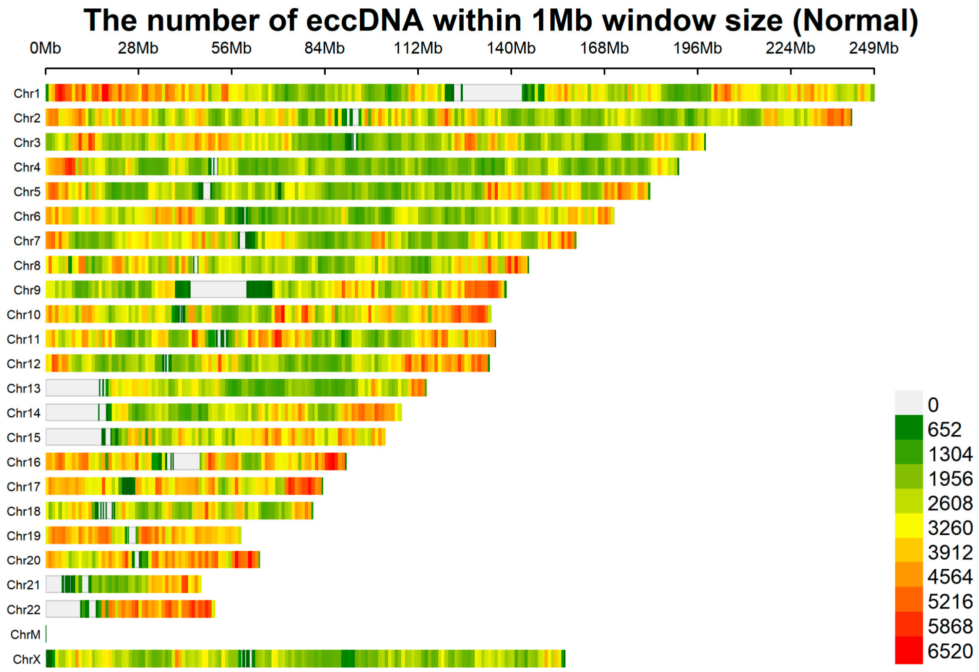

Figure S2: Genome-wide distribution of eccDNA number in 1 Mb genomic windows matched normal tissues.

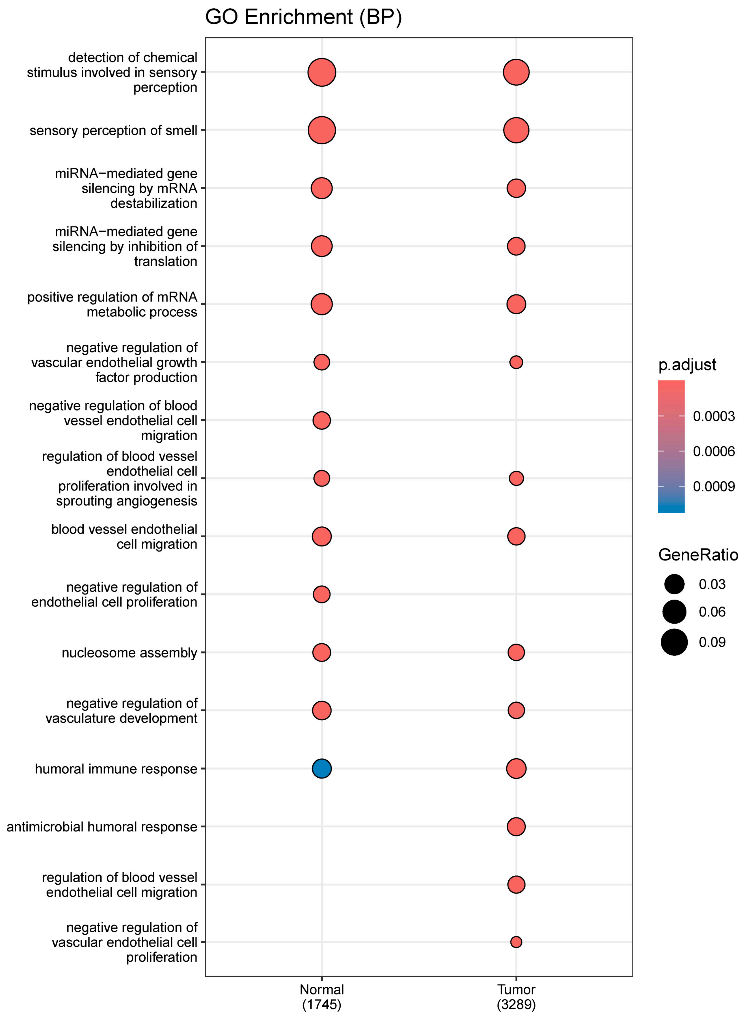

Figure S3: The GO pathway enriched by eccDNA annotated genes in breast cancer tissues (Tumor) and matched normal tissues (Normal).

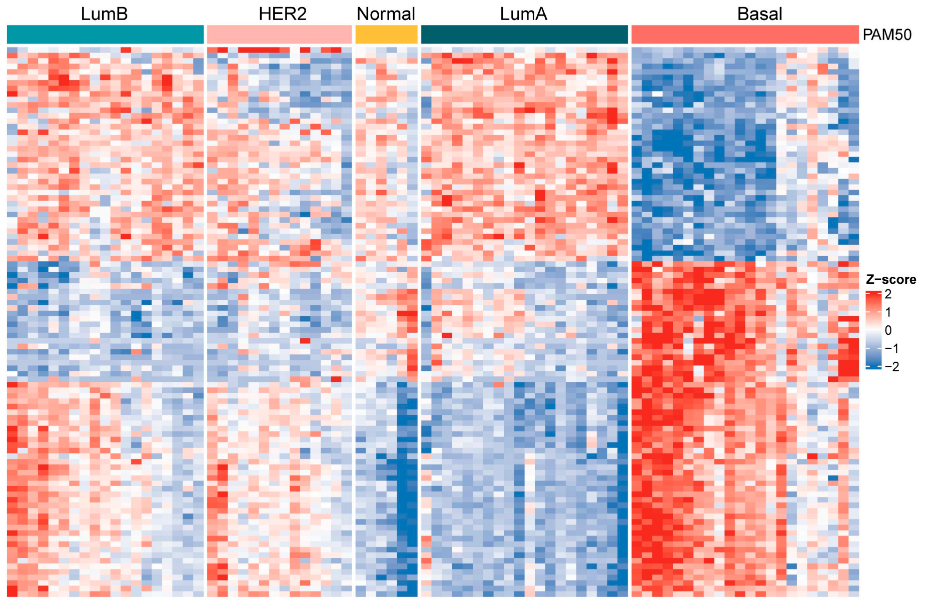

Figure S4: Heatmap of differentially expressed genes (n=100) across breast cancer PAM50 subtypes.

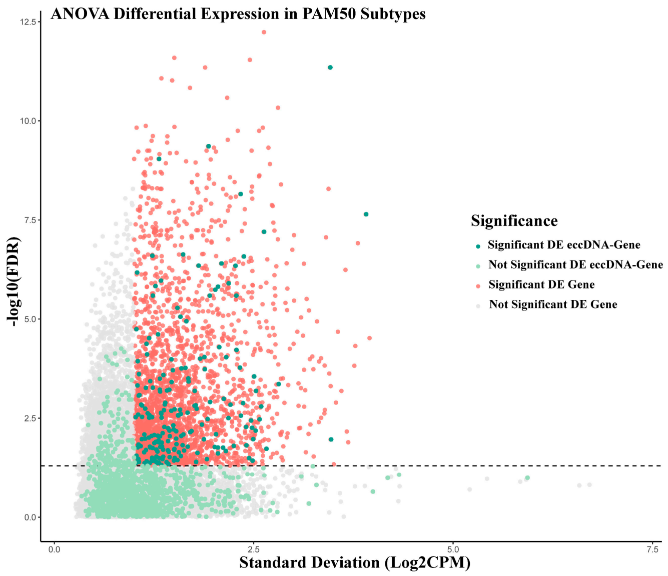

Figure S5: Volcano plots of differentially expressed genes and subtype-specific eccDNA-annotated genes across breast cancer PAM50 subtypes.

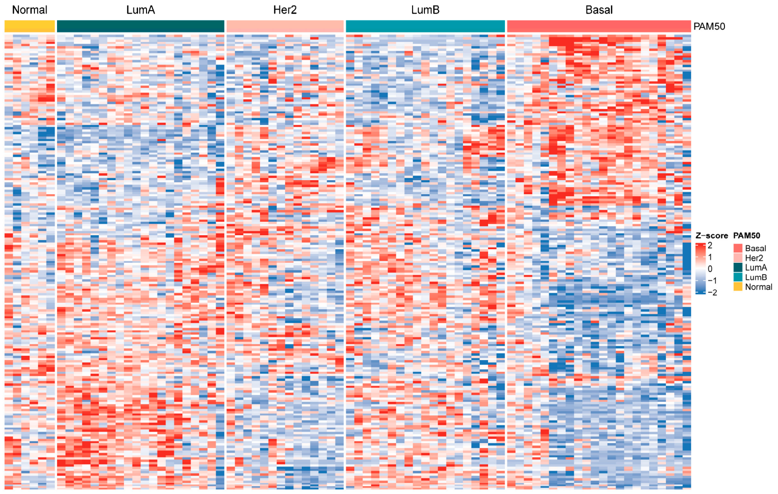

Figure S6: Heatmap of differentially expressed subtype-specific eccDNA-annotated genes across breast cancer PAM50 subtypes.

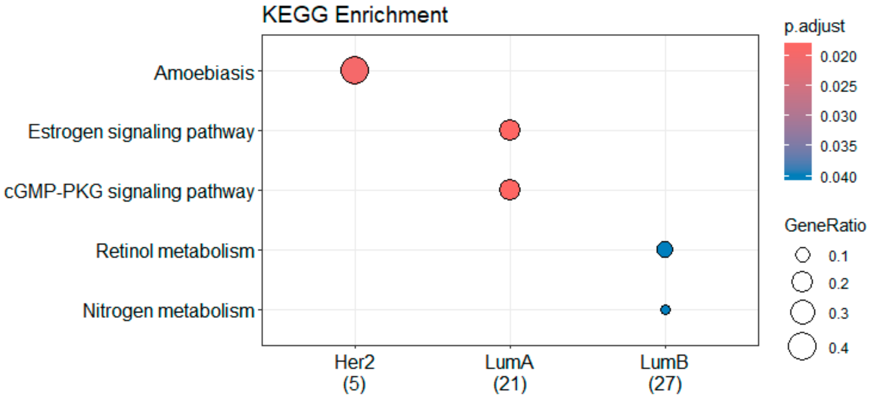

Figure S7: KEGG enrichment of differentially expressed subtype-specific eccDNA-annotated genes across breast cancer PAM50 subtypes.

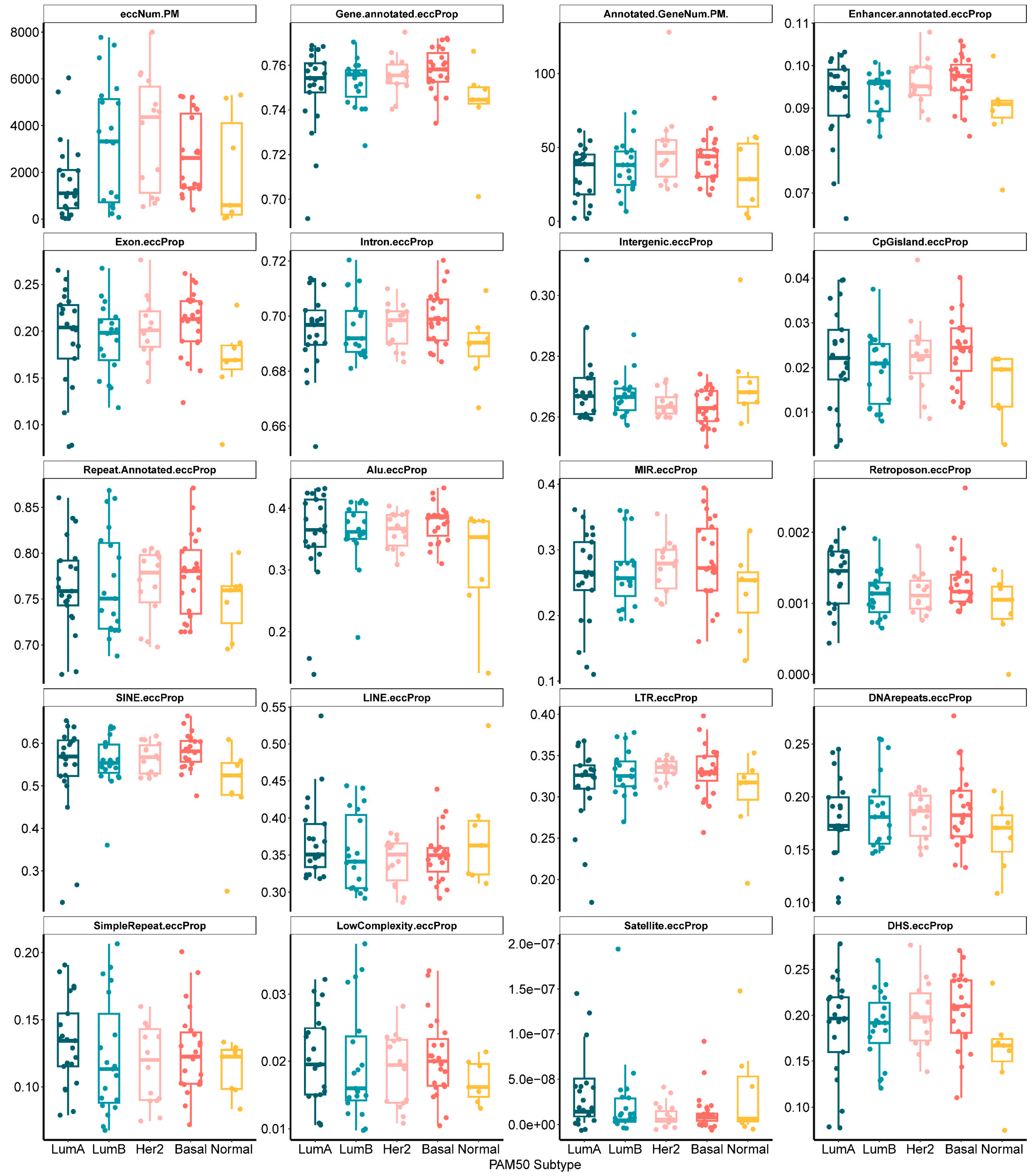

Figure S8: Comparative distribution of eccDNA across genomic elements and repeat sequences in breast cancer subtypes. PM, the number of eccDNA or gene per million mapped reads; eccProp, the proportion of annotated eccDNA.

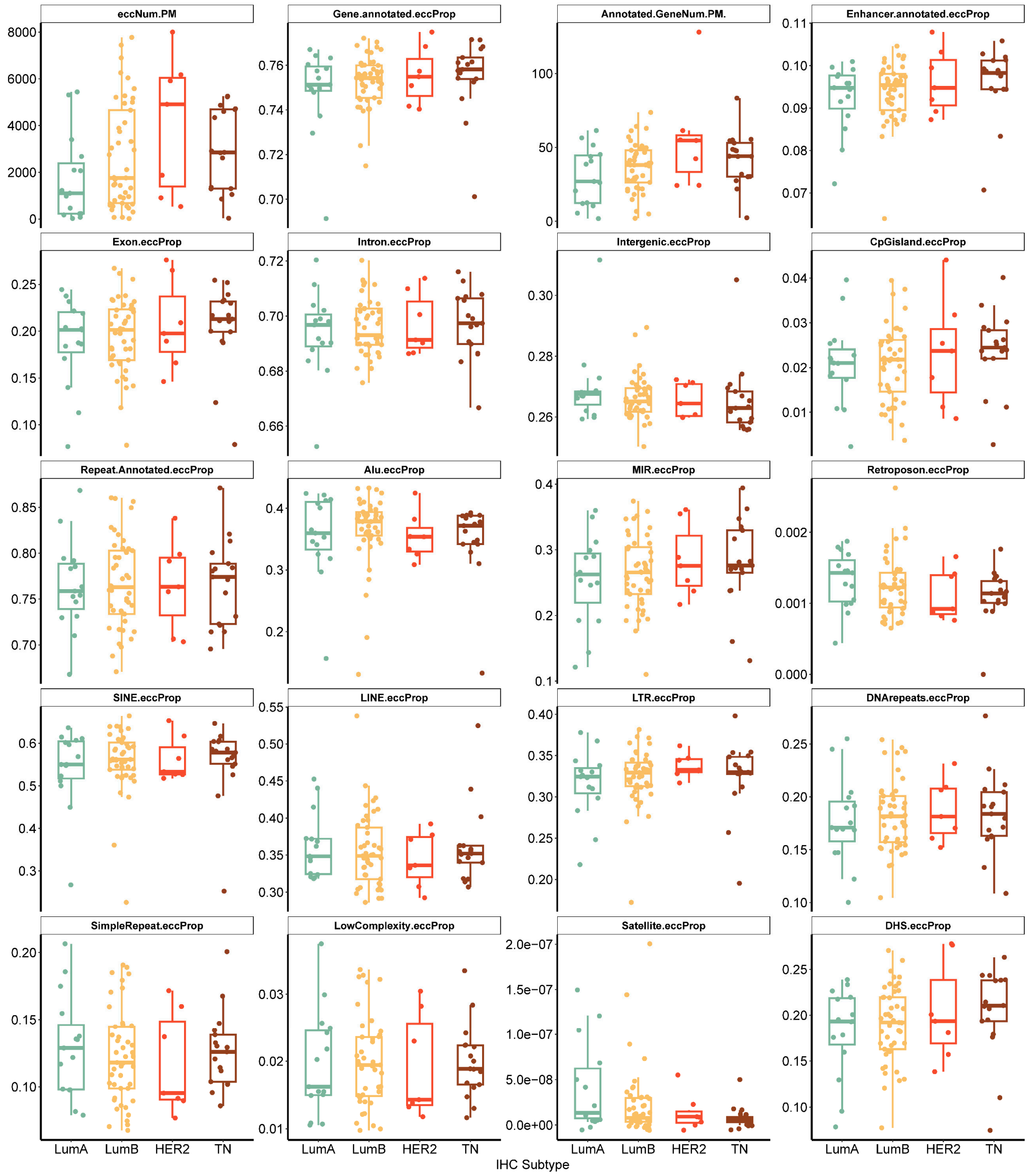

Figure S9: Comparative distribution of eccDNA across genomic elements and repeat sequences in breast cancer IHC subtypes. PM, the number of eccDNA or gene per million mapped reads; eccProp, the proportion of annotated eccDNA.

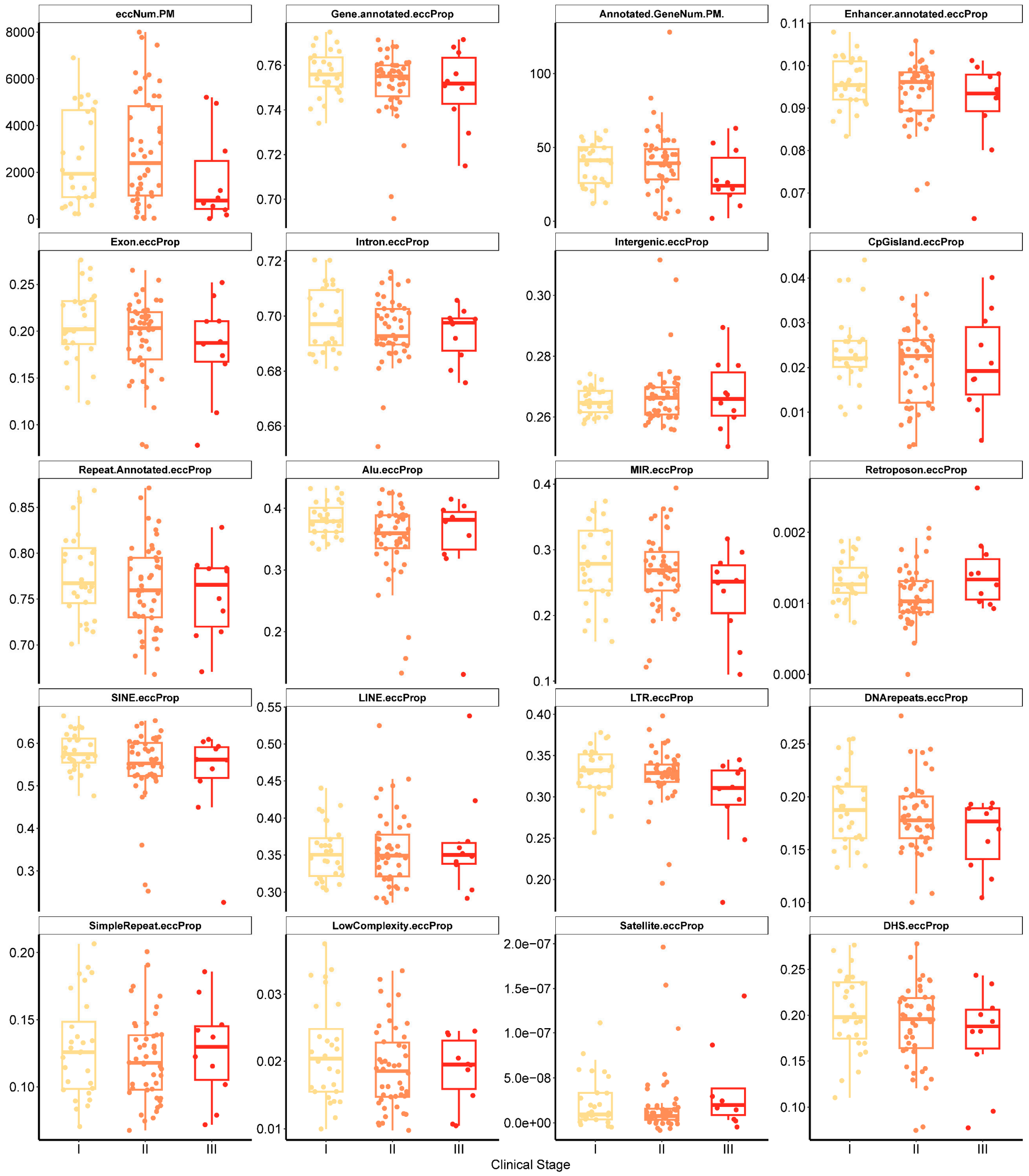

Figure S10: Comparative distribution of eccDNA across genomic elements and repeat sequences in breast cancer samples with different clinical stage. PM, the number of eccDNA or gene per million mapped reads; eccProp, the proportion of annotated eccDNA.

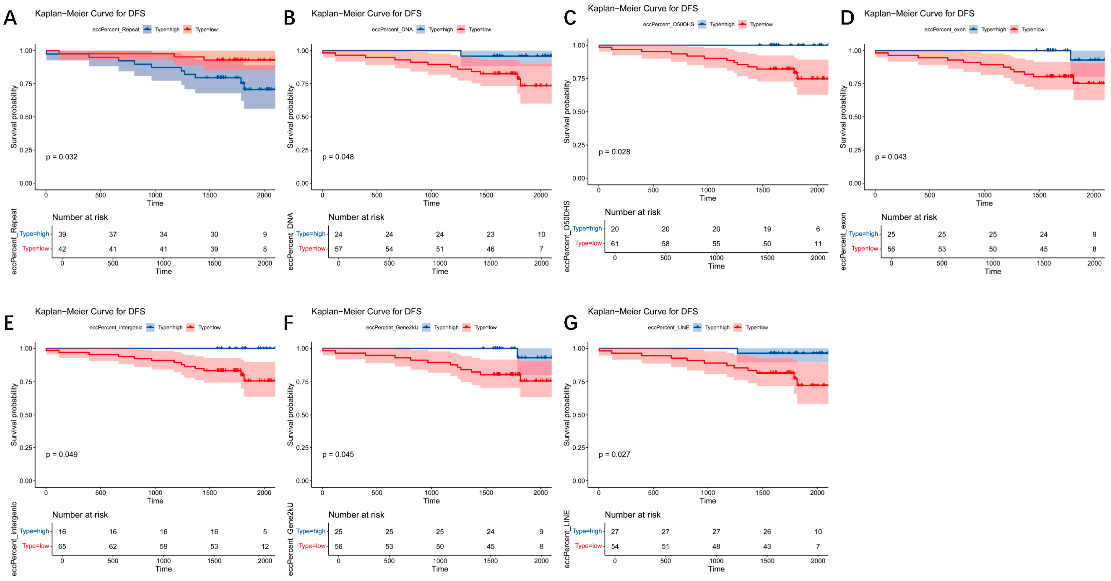

Figure S11: Kaplan-Meier curves of 7 eccDNA features for DFS in breast cancer.

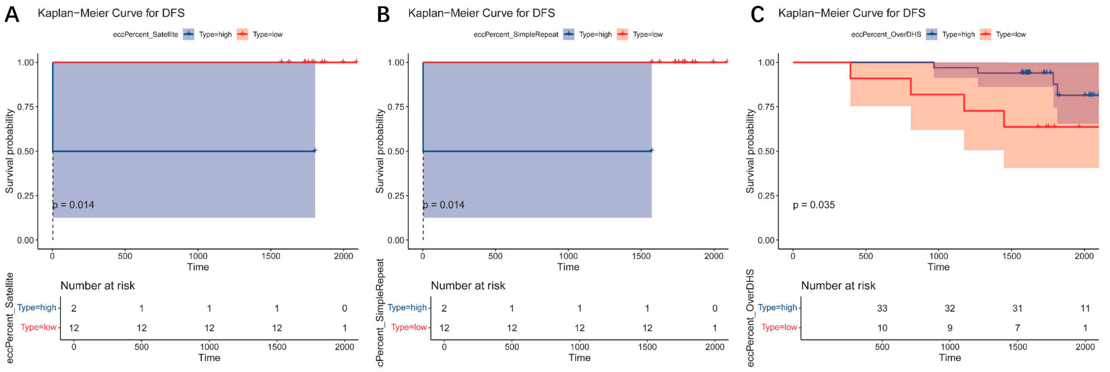

Figure S12: Kaplan-Meier curves of 2 eccDNA features for DFS in Luminal-A breast cancer (A, B) and 1 eccDNA for DFS feature in Luminal-B subtype (C).

Supplementary Table S1. Summary of eccDNA annotation and sequencing on breast cancer tissue and noncancerous adjacent tissue

ID	Label	Mapped Read	eccDNA	Number of annotated eccDNA																					
				Gene	Exon	Intron	intergenic	Gene 2kU	Gene 2kD	CpG island	CpG 2kbU	CpG 2kbD	Enhancer	DHS	Repeat	Alu	MIR	DNA Repeat	LINE	Low Complexity	LTR	Retroposon	Satellite	Simple Repeat	SINE
N01	NAT	111480006	147498	110934	28622	101888	39223	12867	12848	3858	6252	6212	13812	10553	113101	46522	26230	18267	38132	2088	35207	170	434	13210	65562
N09	NAT	85148207	130942	96672	16924	89702	35423	8782	8551	1549	3539	3543	10845	3580	93701	30357	15760	12227	33669	1045	24940	106	477	8376	43592
N10	NAT	100176565	436087	326789	80763	301971	116853	35960	36014	8724	15418	15608	39693	29874	335999	122124	81979	56954	109388	5084	111129	367	1079	32602	184788
N12	NAT	111085399	55012	40952	7934	37614	14597	3979	3982	904	1710	1709	4768	1560	39452	16164	6458	5124	11742	426	10666	47	187	2916	21494
N15	NAT	68672309	11503	8367	1167	7908	3190	652	639	65	201	217	846	245	8103	2238	1108	1027	3301	120	1979	8	58	770	3203
N24	NAT	72666722	8146	5892	778	5547	2310	415	423	21	128	142	553	159	5556	808	839	657	2716	70	1246	3	32	547	1577
N26	NAT	90597195	238782	175277	28045	162439	65144	14864	15131	2468	5769	6001	19468	4599	165039	51232	24675	20571	57233	1556	43079	155	821	12408	72878
N29	NAT	103057123	1602	1146	209	1071	461	115	91	26	46	42	137	78	1195	345	192	141	359	18	316	1	7	234	500
N30	NAT	107602891	269102	201978	47618	186257	71216	21913	21845	5977	10439	10523	24761	14602	201719	75725	44478	31355	65617	3067	60675	279	870	19744	110172
N31	NAT	111448708	734043	554079	138188	510810	191220	60777	60911	14163	28040	28359	70495	49221	552101	201073	138226	89878	176603	7291	175947	527	2007	49076	307375
N33	NAT	157243024	4302	3080	398	2849	1237	255	254	41	79	91	330	41	2779	667	319	344	1029	20	622	1	21	233	967
N35	NAT	144092729	534666	398804	84384	363305	141301	40506	40291	10783	19621	19505	47853	17599	366955	134074	66270	48050	108390	3807	106774	448	1626	25857	188671
N36	NAT	86228008	253436	187725	36565	175650	68584	17331	17186	2178	5861	6115	21145	11487	192611	70488	39673	31913	63610	2531	59013	164	805	17191	102182
N39	NAT	82247915	243947	182634	41673	167879	64808	19627	19793	5112	8959	9232	21811	12397	179918	70402	37327	27200	56181	2722	54309	216	671	16508	99165
N40	NAT	24584463	434	315	54	296	126	25	31	2	7	11	28	10	322	58	66	49	143	4	84	0	3	59	118
N41	NAT	80700567	504531	376112	79413	348148	134710	37245	37281	8426	15632	15658	44755	23985	373633	134868	76334	57556	119403	4671	114545	345	1361	31195	195949
N42	NAT	70984586	396269	298488	72319	271559	103294	33138	33114	10493	16706	16673	37088	9781	284951	107165	58784	39654	83887	3322	85634	298	1050	20901	154432
N43	NAT	67925203	790	551	65	521	242	33	34	7	22	16	65	9	553	104	56	37	263	11	90	1	8	88	158
N47	NAT	28536247	79	58	12	56	20	6	4	4	4	2	5	6	52	9	11	7	17	3	12	0	0	12	20
N49	NAT	143496479	385990	288646	63145	266060	102501	29568	29749	6482	12700	12928	34605	18697	284758	109007	58769	43918	87268	3658	86294	285	1008	23642	155343
N50	NAT	90153485	145608	109318	25682	101465	38673	11599	11585	2194	4694	4817	13311	9415	113202	45619	26634	19624	37188	1956	35739	127	377	12095	65407
N52	NAT	102223337	3205	2366	400	2186	825	205	178	41	77	85	273	56	2042	673	307	227	568	12	574	1	13	103	956

N55	NAT	118503670	60638	44660	7180	40783	16294	3794	3778	656	1557	1571	5136	730	39136	13212	5586	4608	10857	279	10754	32	246	1717	18311
N56	NAT	71389783	270898	203153	47413	187219	71933	22048	21936	5569	9898	9974	24649	15502	204916	81026	44595	32011	65406	3141	63395	215	722	19532	114955
N58	NAT	143103035	377335	282637	65702	260191	100382	30603	30378	8761	14070	14168	34087	20169	280505	106921	60182	43662	89543	4173	84692	390	1180	26243	153467
N64	NAT	97605270	482210	357862	73141	332044	130016	34724	34633	6077	13580	13494	42360	21383	361125	129715	74250	56043	114619	4081	113662	315	1262	29066	189396
N66	NAT	97378032	222828	166024	36574	152205	59600	17364	17312	4666	7951	8112	19784	9214	160208	59651	32528	23297	46996	1987	48103	202	700	12389	85654
N70	NAT	62857059	211839	158318	36628	146283	56765	16859	16728	4174	7355	7572	19072	11941	159334	60416	35878	26283	51262	2432	50164	178	578	15180	87988
N71	NAT	60629242	307761	231761	58679	211723	80887	26666	26452	7937	13181	13009	29349	18816	228113	86063	53741	36018	74461	3682	71547	239	835	23114	126873
N74	NAT	59461166	49942	37823	10740	34966	13292	4661	4792	1505	2227	2326	4729	4805	39704	16872	10838	7696	14529	1017	12907	80	142	5869	24105
N75	NAT	89193370	426648	318862	71434	293857	113474	33389	33190	7746	14582	14584	38446	21673	312782	112901	67355	48280	95442	3784	98220	311	1159	25156	166479
N77	NAT	107249192	513186	382082	71937	355021	136229	35334	35239	4649	12948	13199	44928	18903	375276	136411	75505	57133	114541	3927	113693	280	1579	27197	197839
N79	NAT	78128255	286282	212130	40798	196354	77053	20471	20042	3543	8221	8165	24844	19100	208815	75247	38902	30346	64082	2164	62300	177	813	15521	107376
T01	T	109750080	25362	19358	6026	18270	6787	2431	2439	531	887	970	2430	3621	22025	9086	7926	5615	9700	826	8323	32	84	4549	14023
T02	T	63043802	40258	30445	9179	28668	11034	3879	3840	804	1492	1409	3797	5259	34641	14906	11564	8377	14804	1116	12654	60	120	6609	22183
T03	T	83304055	438149	334513	92528	304984	112057	40114	40063	12401	20246	20731	43484	32257	320371	109539	87196	53981	101733	5375	105150	322	1341	32603	176661
T04	T	76127734	157319	120674	38454	110736	40880	16203	16175	5581	8451	8395	15881	17858	123547	49496	38522	23665	42788	3080	40782	189	532	16991	75501
T05	T	84589238	39883	30199	8933	28428	10821	3794	3667	810	1412	1465	3852	5252	34287	14058	11914	8452	14299	1118	12732	43	110	6483	21765
T06	T	78425121	405829	310967	92452	287844	104630	38259	38328	8901	17021	17157	41499	46143	324995	123080	106937	66836	117984	6949	114803	412	1162	43259	197858
T07	T	90745673	20556	15374	3513	14138	5488	1691	1691	468	785	790	1813	1014	15474	6561	2978	2276	4941	240	4385	29	74	1884	8799
T08	T	70118957	37535	29085	10360	26648	9756	4305	4132	1653	2327	2438	4050	5169	29708	11353	10539	6134	11205	838	10230	42	96	4750	18337
T09	T	68246113	355721	266648	58773	243981	93230	27860	28124	7473	13313	13254	32866	14715	254126	97933	48857	34412	74104	2655	75436	260	947	18278	137189
T10	T	81554333	375402	283132	74819	261570	99615	32526	32651	8235	14607	14892	35443	30476	289488	107617	78407	53235	98795	5465	96945	344	1021	33082	165154
T11	T	77221287	136024	102730	30014	95905	36679	12109	12105	1988	4340	4269	13109	15843	112293	41391	39492	27274	43490	2926	42848	99	340	17927	68847
T12	T	112117801	168410	125537	26579	116525	45246	12484	12737	2608	5228	5202	14831	8345	127894	49665	25743	19759	43953	1920	37499	155	558	13040	69568
T13	T	115397631	475905	358110	86248	328317	125175	39266	39624	10522	18427	18619	44226	26830	353382	137769	77158	53701	109660	4964	110030	436	1336	31649	196819
T14	T	97416092	205671	156577	45779	144876	53918	19265	19325	4741	8625	8850	20506	21433	165660	64537	49927	33499	62875	3905	57138	206	538	24421	99136
T15	T	85273845	2871	2013	227	1914	876	134	150	8	38	40	203	44	1997	265	262	217	1048	26	390	0	32	258	504

T16	T	90760319	77716	58796	16849	54413	20744	7150	7214	2037	3353	3445	7669	7727	62227	24181	17579	11865	22580	1377	21078	64	283	9162	36455
T17	T	93979518	37236	28603	7848	26036	9320	3486	3527	1239	1840	1903	3626	2528	27444	11383	6508	4329	8314	537	7920	72	238	2791	16264
T18	T	121060182	96141	73060	20689	67557	25103	8887	8846	2300	4131	4030	9464	8745	76459	31169	21311	14666	27394	1748	24793	113	361	10580	46016
T19	T	89779594	81011	60985	15293	56615	21657	6910	6819	1415	2827	2795	7492	6721	63492	25197	16903	12003	21393	1299	21149	80	237	7767	37239
T20	T	79525884	6023	4525	1220	4210	1618	527	494	113	221	213	587	501	4542	1348	1359	906	1688	92	1535	2	24	615	2375
T21	T	86572211	98177	73681	18685	68734	26494	8231	8089	1592	3013	3087	8969	8241	79375	30875	22495	16312	27683	1542	26848	78	322	10236	46989
T22	T	115772378	384219	288764	75879	264802	102426	33033	32820	8284	14826	15344	36421	29528	290592	101900	74577	50297	98661	4641	97112	274	1125	31503	158222
T23	T	85030536	150831	114850	35261	105825	39750	15234	14841	4055	7273	7219	15334	15949	119979	47253	37190	23729	43981	2787	40398	161	476	17353	72977
T24	T	76657613	376524	279268	62599	259897	102525	28097	28009	4208	10501	10508	33592	20912	287404	95912	72764	52135	95618	3966	95263	237	1075	27395	153176
T25	T	181404531	240982	186075	63072	173568	62894	25275	25168	6216	11030	11242	25206	38407	204744	88646	76591	49464	83765	6723	74652	334	784	37909	136049
T26	T	81864122	14377	10795	2678	10035	3852	1168	1219	249	536	524	1357	1135	11260	3586	2810	1908	3922	168	3489	19	44	2092	5756
T27	T	95012533	122880	93160	28464	87996	33269	11309	11200	1524	3607	3475	11995	18077	107051	41596	42199	29605	46995	3586	42613	95	302	21479	69240
T28	T	139754036	76549	58249	17105	53051	19976	7346	7319	2789	3960	3986	7464	6068	56887	21744	15111	9821	18387	1111	17804	117	297	6691	32699
T29	T	111026331	135809	99084	15333	92387	37633	8452	8313	1432	3206	3140	10884	2517	96446	31390	13851	11785	35528	1031	23929	95	556	7622	43362
T30	T	93222525	437156	332899	100473	308816	115121	41978	41966	10363	19027	19094	44593	49501	355543	133044	117180	75145	128929	7959	125934	414	1801	47140	215770
T31	T	80169070	1610	1151	126	1088	466	65	77	6	19	23	103	15	1080	141	119	113	581	21	186	1	11	158	244
T32	T	97055814	126482	95818	26600	88419	33253	11505	11454	2994	5325	5248	12336	11237	99020	38881	26264	17798	34752	1981	32721	130	425	12888	57568
T33	T	138438719	145899	107098	18054	99709	39995	9384	9238	1628	3626	3571	12162	3775	105258	35973	16877	14015	37345	1225	27028	120	753	9042	50154
T34	T	115901192	78780	60322	18744	55283	20476	7804	7911	2393	3831	3835	7849	8284	61984	25016	18366	11744	22302	1482	20907	112	180	8480	37760
T35	T	78639175	36755	27880	8145	26149	9849	3326	3327	667	1212	1173	3484	4796	30687	11201	10740	7518	12729	918	11283	39	111	5369	18468
T36	T	87774063	462982	344204	67791	319395	123818	32096	32425	4969	12019	12499	41156	20103	339728	120868	71014	54735	107908	4121	106572	275	1255	28763	177585
T37	T	90567408	393263	303353	100079	278377	101050	40091	40421	13340	20714	21013	41616	51437	308404	106362	111768	63846	110401	6892	107446	335	1510	42740	186377
T38	T	71369395	207607	160174	52312	146510	53168	21222	21428	8331	11611	11875	21009	24509	162063	62217	51333	31286	57053	3921	53266	231	788	22996	97850
T39	T	74879429	107866	81987	25113	75806	28699	10631	10612	3099	5074	5143	10676	12180	86493	36732	26797	17364	31187	2187	28909	166	380	12568	54467
T40	T	82456732	508905	376770	74427	351815	138064	35042	35342	4375	12089	12198	44420	24685	385781	136668	83606	65730	129669	5102	122201	294	1310	35285	203025
T41	T	80597372	44454	32915	7736	30990	12312	3513	3529	571	1298	1325	3923	3527	36818	13931	9327	7145	15591	900	11475	52	198	6280	20640

T42	T	116182637	105422	79160	20825	73853	28505	8962	8923	1873	3494	3550	9700	8447	84222	29791	24296	17606	31260	1935	29206	116	351	11547	47535
T43	T	70186180	1704	1178	131	1112	531	87	76	4	21	22	123	16	1138	178	138	114	515	12	248	2	12	133	304
T44	T	89104810	252647	190465	47821	173369	66141	21334	21492	6049	10582	10758	23901	14393	180489	65488	42875	28862	57424	2909	56318	208	928	18741	98503
T45	T	24013142	141951	107503	29670	97432	36880	12678	12646	3605	6424	6363	14121	10158	99860	30820	27520	16059	30694	1428	33216	92	374	8957	52691
T46	T	115783562	127874	97226	28000	89777	33505	11908	11827	3330	5855	5772	12738	12350	101575	41422	29251	19249	37819	2607	32830	148	639	15706	61657
T47	T	121182331	942071	698210	133431	646736	253232	64594	64415	10332	25260	25612	82214	34377	674401	235994	131236	101808	205662	6582	203195	494	2765	45692	344561
T48	T	43248885	204150	155391	43469	140824	52719	18838	18842	6165	9858	9869	20058	15176	145822	45239	39417	23632	44760	2148	47778	145	699	13961	76680
T49	T	76846160	261189	195801	48044	180269	69520	21452	21820	5490	10051	9946	24219	16196	195089	70195	48020	33342	62575	3166	63277	242	823	19053	107311
T50	T	84016461	248368	185114	42623	173184	67214	19096	18790	2998	6724	6702	21658	17549	199944	77040	47540	36507	69943	3248	67837	206	544	21793	112355
T51	T	140052773	524769	395836	94962	360678	136618	43471	43481	12644	21540	21654	50396	27736	375666	142121	80198	55045	109529	4596	115007	460	1464	29725	205342
T52	T	98145580	677091	501353	94512	464459	182314	46302	46383	6442	17292	17400	58825	24005	491549	177820	94404	72941	148836	4901	149106	360	1953	34683	255057
T53	T	80023144	640205	483279	121188	439603	166951	53987	53606	15177	26367	26637	60676	37076	452346	147408	107281	68789	132247	5331	148397	396	1930	34803	234104
T54	T	98391608	487408	368561	102565	337235	128963	43900	43686	12196	20460	20509	47794	41341	365806	130186	102325	67358	124778	6851	126141	416	1449	42186	205585
T55	T	81344306	491290	363327	73035	340169	134026	33896	33678	3507	10957	11066	42127	26903	379358	128091	90486	68895	132971	5612	126450	273	1496	38977	199012
T56	T	77780857	5630	4076	666	3834	1616	318	352	53	128	128	469	227	3873	738	802	588	1718	60	1045	3	27	457	1396
T57	T	70460780	352342	266307	70957	241971	92219	31375	31253	9265	15788	15807	33607	23301	252582	86709	63416	40398	77334	3693	82142	256	1059	22796	135895
T58	T	149818930	391386	294512	78278	272941	105042	34328	34404	8624	15275	15235	36958	32247	308669	116618	85216	59040	109823	6413	101769	426	1688	38870	178448
T59	T	100049384	523409	397990	113571	361512	135599	48091	48206	15163	25083	25039	51751	40933	378337	131809	106433	64803	118728	6255	124442	395	1778	38837	212350
T60	T	100315626	209767	160330	48593	146156	54396	21131	21055	8309	11437	11643	20784	18710	159146	65907	41782	26834	54451	3470	49628	285	598	20490	94971
T61	T	71692582	390175	295545	79572	269313	101657	34811	34866	9895	17074	17267	37386	27244	285328	98782	75793	48273	90798	4294	93074	285	1310	28062	156976
T62	T	124443327	483742	367614	97680	333682	124440	42979	43194	13073	21972	22324	47437	31257	347494	127227	85307	54628	105624	5185	109173	416	1626	31847	192404
T63	T	77491972	213274	160718	44273	147066	56827	19343	19402	6065	9437	9600	20131	16424	159579	59222	41435	27510	53239	3023	52571	200	683	18371	89856
T64	T	85930000	25797	19123	4365	17794	7092	1930	1923	299	729	729	2306	1675	19719	5611	5036	3364	7692	392	6386	14	93	2415	9538
T65	T	79156765	148298	113955	39313	105847	39216	16122	16184	4711	7804	7916	15303	21897	124293	52775	44889	28765	48730	3785	44972	206	473	21337	81203
T66	T	80708158	428858	321326	80421	296680	114993	35852	35474	9383	15847	15832	39257	29124	327433	115615	83034	57437	106402	5075	108654	344	1093	32014	179376
T67	T	92894303	265000	203349	63363	188882	68775	25665	25917	6673	11672	11940	26850	32662	217540	84327	72861	49233	87402	6174	76075	292	806	36457	134115

T68	T	102921129	501371	385224	116953	354501	128337	47218	47492	12257	22511	22874	51525	55742	388116	127609	134790	79376	134460	7306	137238	347	1536	46831	227562
T69	T	117727580	382963	289967	79795	264956	100625	34443	34122	9760	16055	16397	36925	29768	282482	101442	76679	51119	94109	5152	93981	371	1205	31977	158250
T70	T	79662225	242222	181933	44093	167213	64166	19916	20103	5306	9333	9466	22275	14404	180807	69237	42027	29162	58379	2925	57255	218	584	17876	101063
T71	T	104831294	7702	5735	1289	5359	2090	575	556	84	200	204	664	556	5851	1515	1616	1108	2357	113	1857	5	26	734	2771
T72	T	56129126	313212	236835	62064	214596	81438	27129	27346	8196	13743	13989	30138	19210	221249	77132	54848	34138	65959	3057	70967	231	997	19055	119925
T73	T	63542239	108439	83183	25704	77330	28173	10627	10695	2801	4821	4913	11022	13417	88957	38428	28719	19337	35325	2536	30678	140	329	15446	56824
T74	T	95021850	90705	69879	24238	64518	24047	10141	10092	3403	5015	5074	9140	12881	73820	29380	25617	16642	29276	2486	26177	141	312	13244	45837
T75	T	86953678	405059	306228	82077	285148	107100	34367	34363	6484	13496	13797	38654	38239	325218	117922	98955	66504	117931	6498	114051	327	1191	40815	189792
T76	T	44885195	43161	33186	11030	30163	11214	4651	4612	1704	2435	2520	4421	5010	32928	11905	10632	6290	11387	778	10939	57	127	4416	19401
T77	T	107273754	798371	597509	130984	559770	212479	57725	57968	6404	19718	20085	71484	50144	619422	185786	171287	120908	218193	9188	211397	404	2651	61225	323380
T78	T	70768719	46058	34923	10671	32769	12414	4208	4392	879	1582	1697	4417	6410	39444	16115	14136	10020	16247	1254	14708	50	136	7267	25226
T79	T	91354490	244469	180244	34203	167149	66696	16841	16991	2647	6670	6732	20813	21683	178395	60833	34202	26267	57727	1934	53260	154	838	14578	89217
T80	T	69920231	437601	328930	79158	299035	114746	35904	36142	9542	17496	17733	40894	9658	305324	102162	68456	44316	87359	3300	97850	285	1362	22812	158299
T81	T	80879561	117642	88734	25410	81366	31469	10967	10859	3387	5254	5388	11396	10146	90970	35292	24333	15988	31481	1893	29718	130	386	11896	52671

Abbreviation: NAT, noncancerous adjacent tissue; T, Tumor; **2kU, 2000 bp upstream; 2kD, 2000 bp downstream.**
